# Correction: Recombinant cyclin B-Cdk1-Suc1 capable of multi-site mitotic phosphorylation in vitro

**DOI:** 10.1371/journal.pone.0330048

**Published:** 2025-08-08

**Authors:** The PLOS ONE Staff

## Notice of Republication

This article was republished on March 28, 2025, to correct errors in Fig 2E and Fig 3C that were introduced during the typesetting process. The publisher apologizes for the errors. Please download this article again to view the correct version. The originally published, uncorrected article and the republished, corrected articles are provided here for reference.

**Fig 2:**
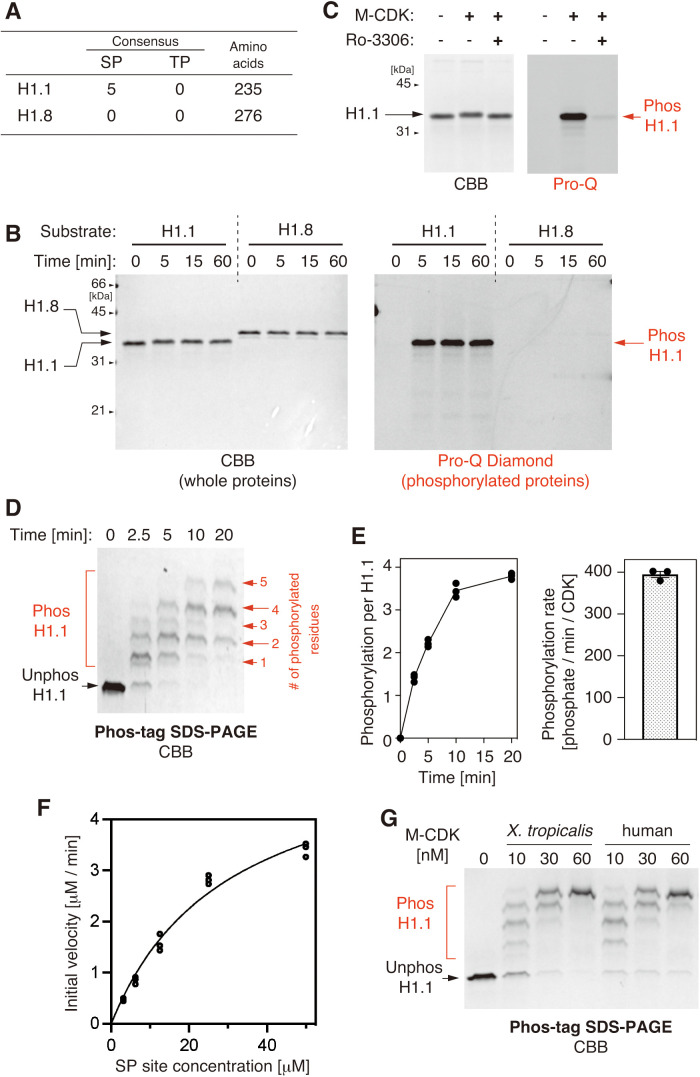


**Fig 3:**
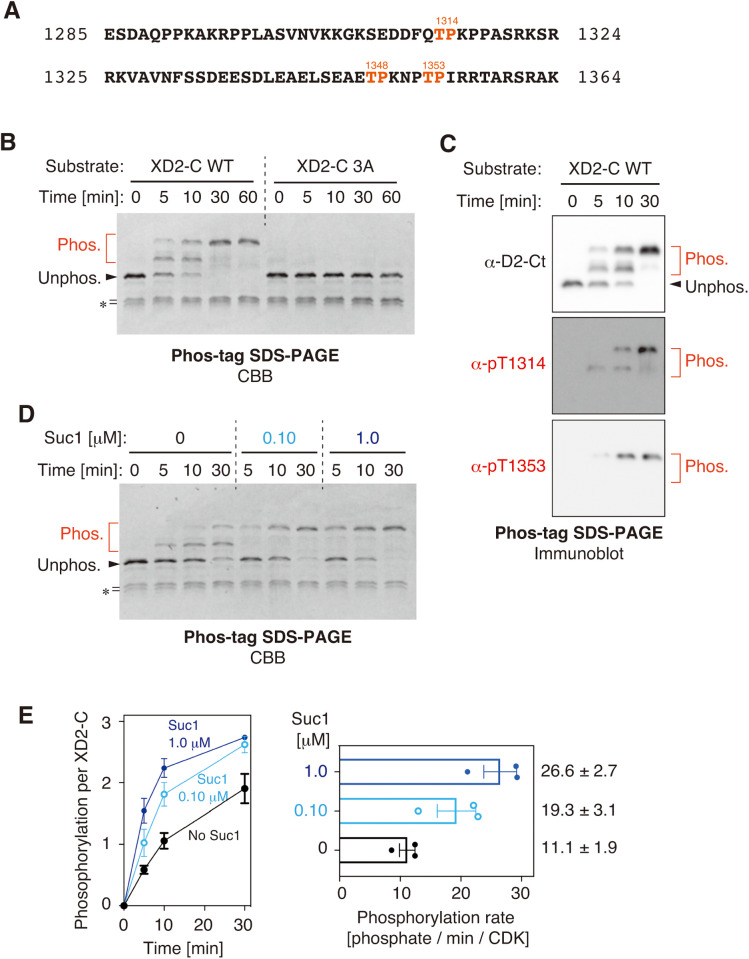


## Supporting information

S1 File Originally published, uncorrected article. (PDF)

S2 File Republished, corrected article. (PDF)
