# Supplementary figures and images for: Recombinant cyclin B-Cdk1-Suc1 capable of multi-site mitotic phosphorylation in vitro
Source: PLoS One. 2024 Mar 25;19(3):e0299003. doi: 10.1371/journal.pone.0299003 (PMC10962838; doi:10.1371/journal.pone.0299003)

Cropped parts are indicated by red dashed rectangles.

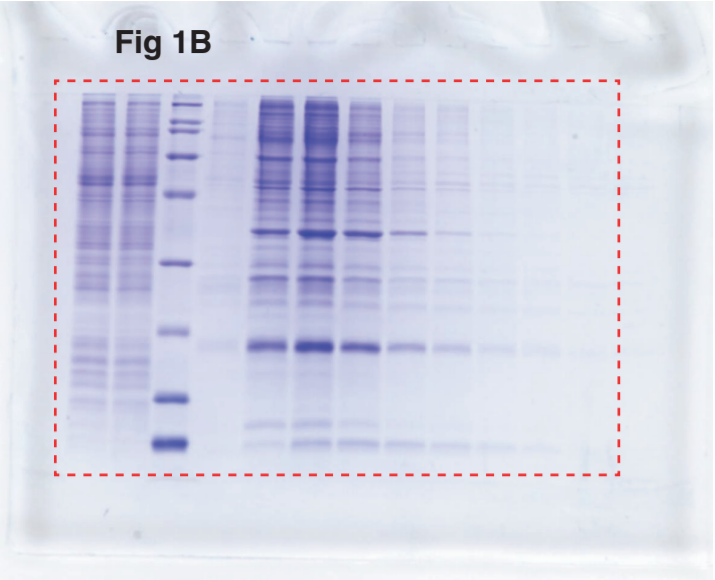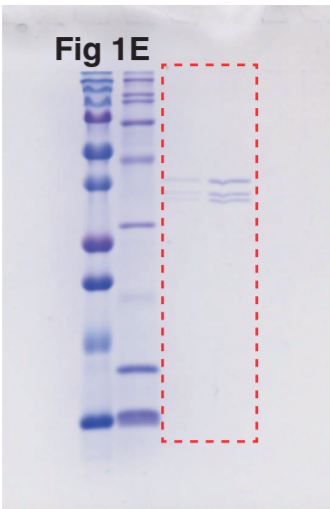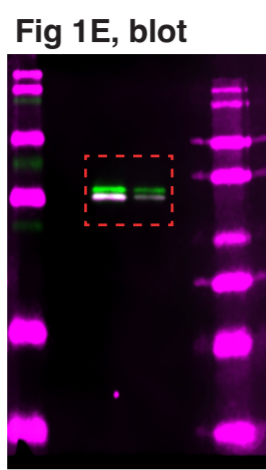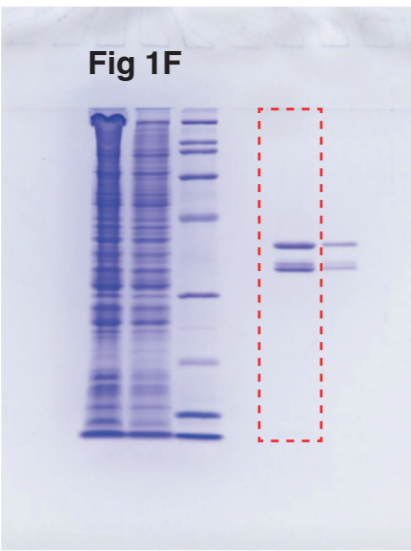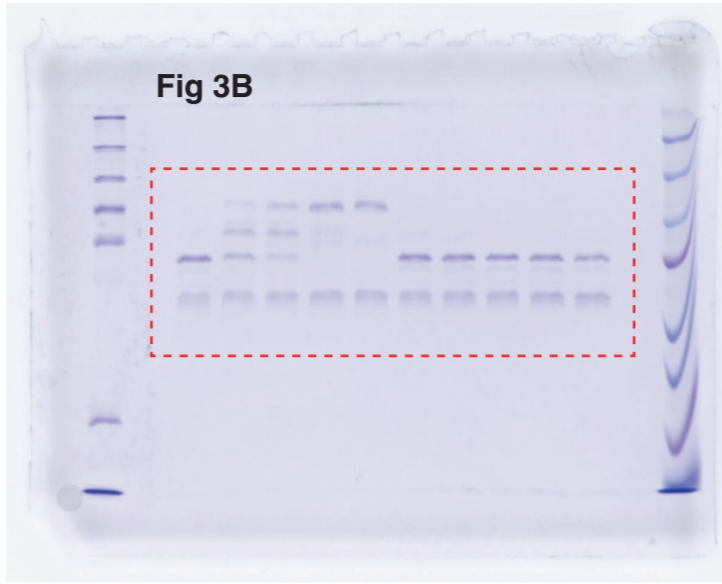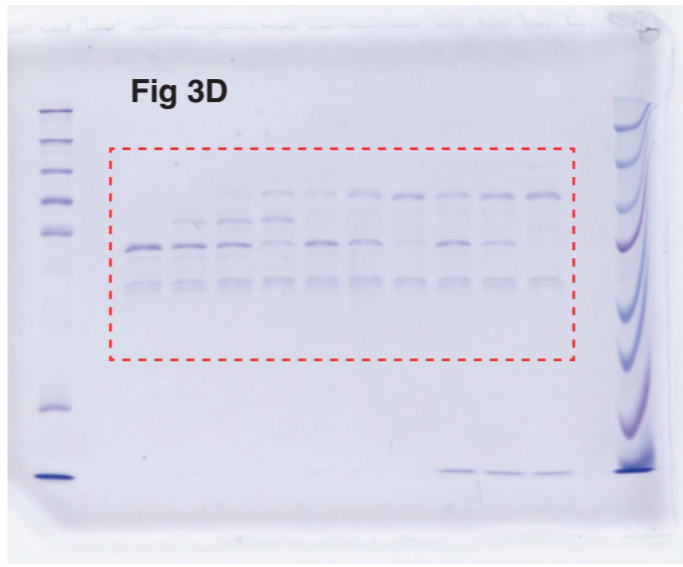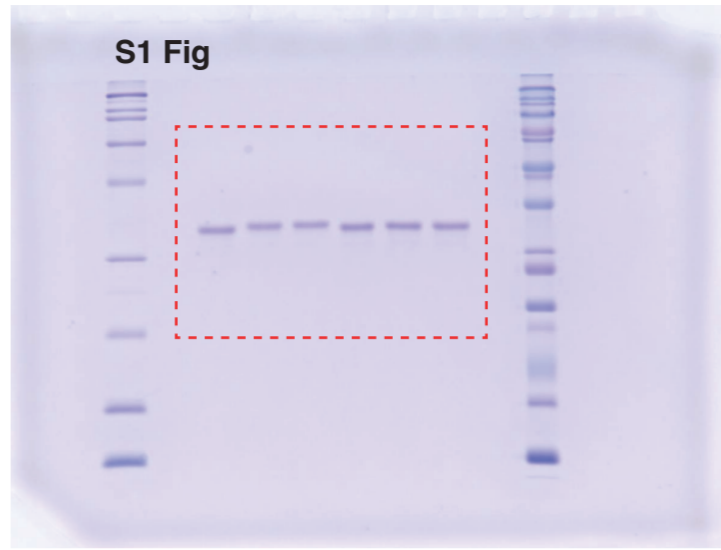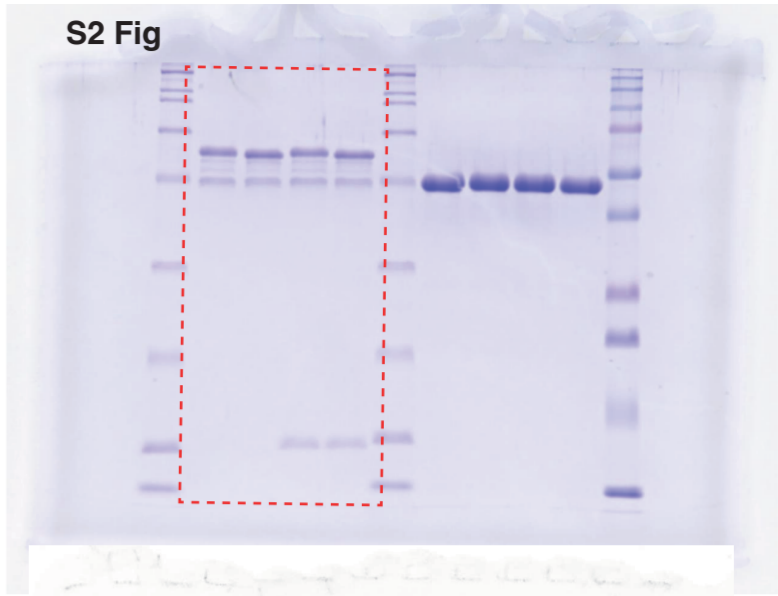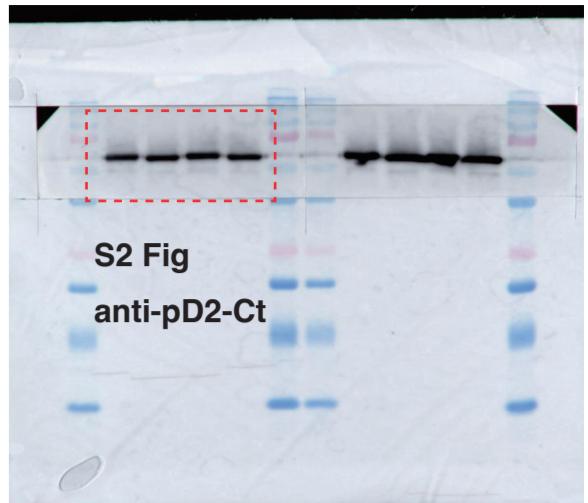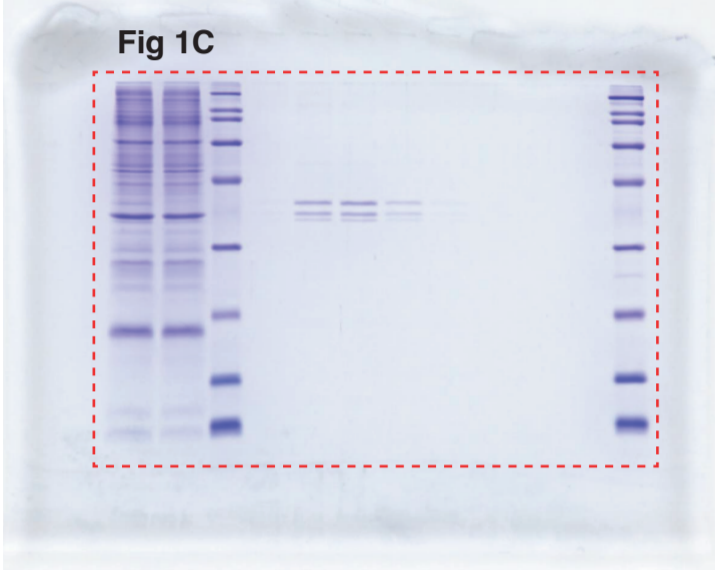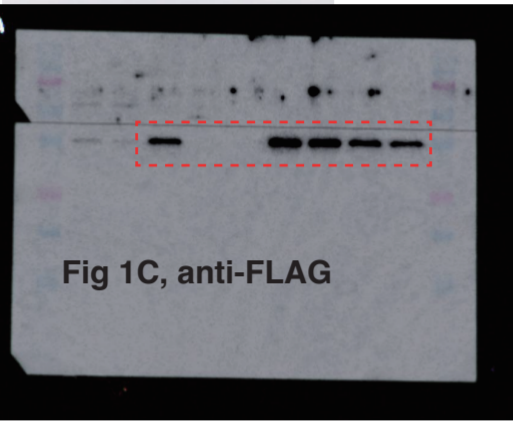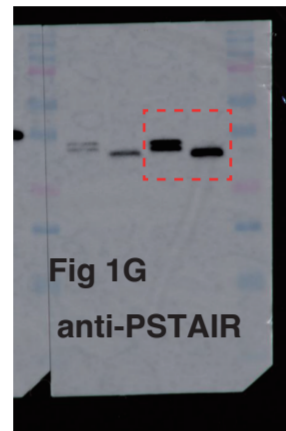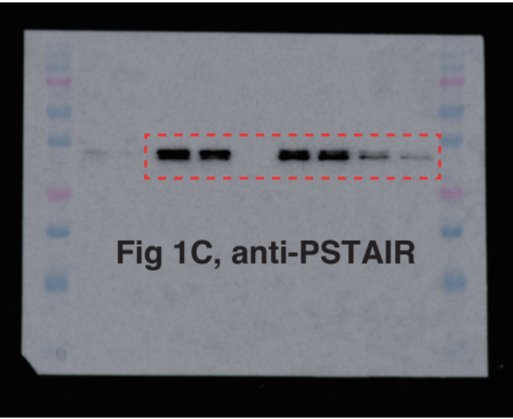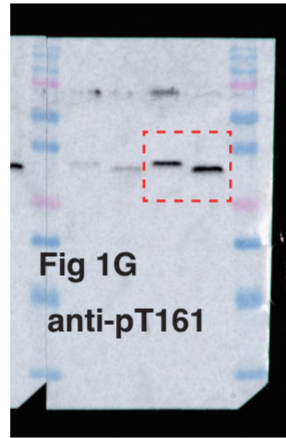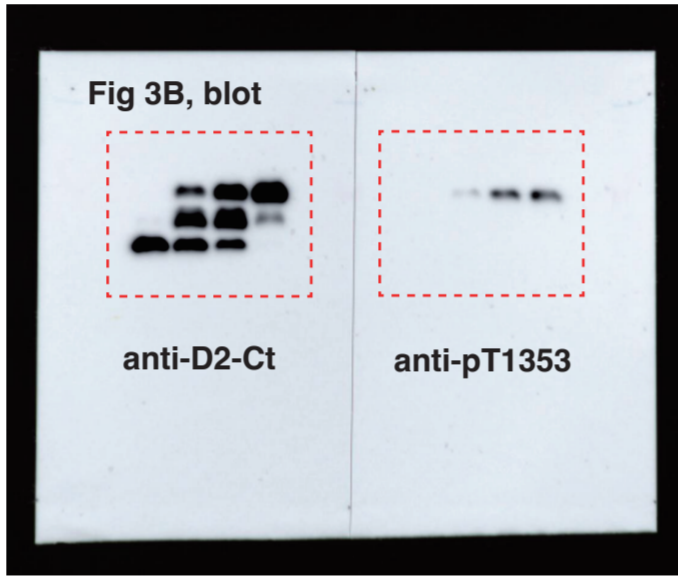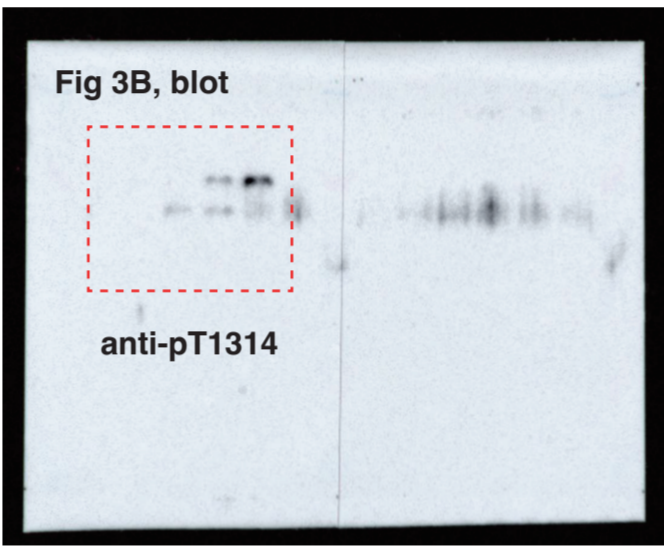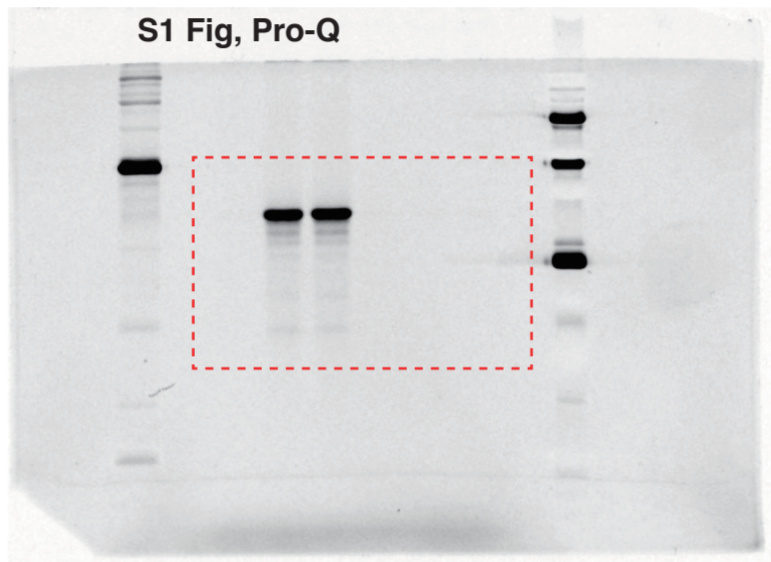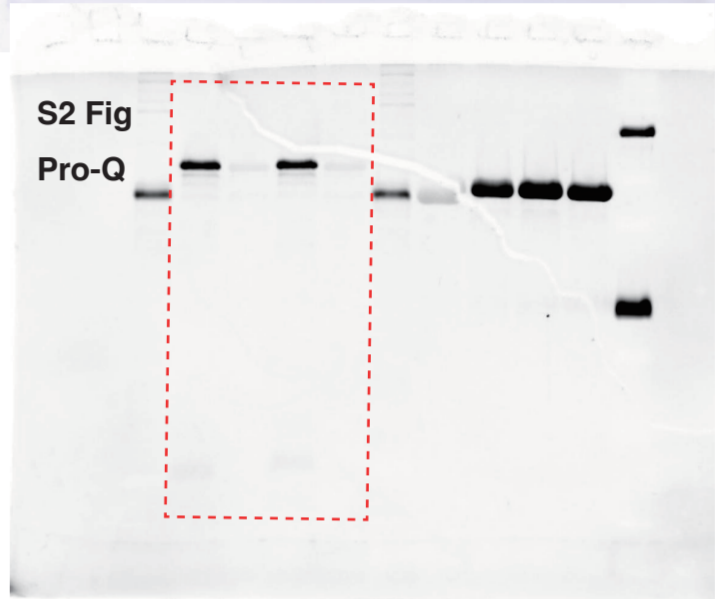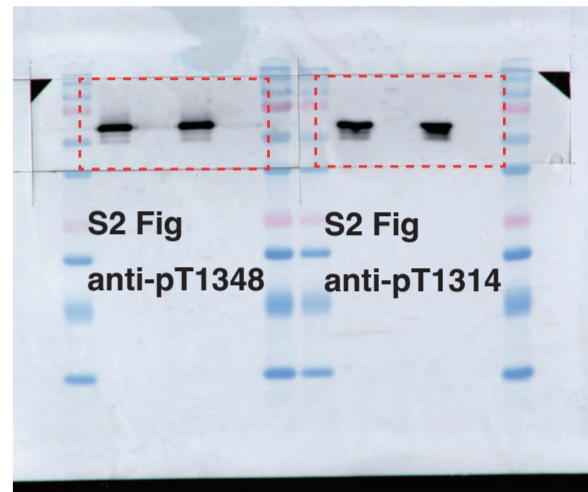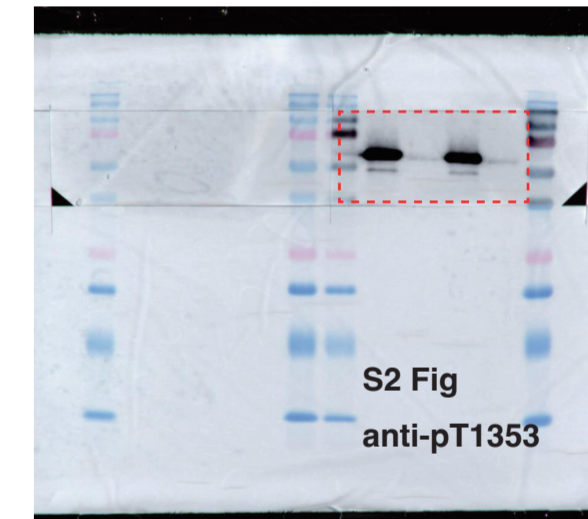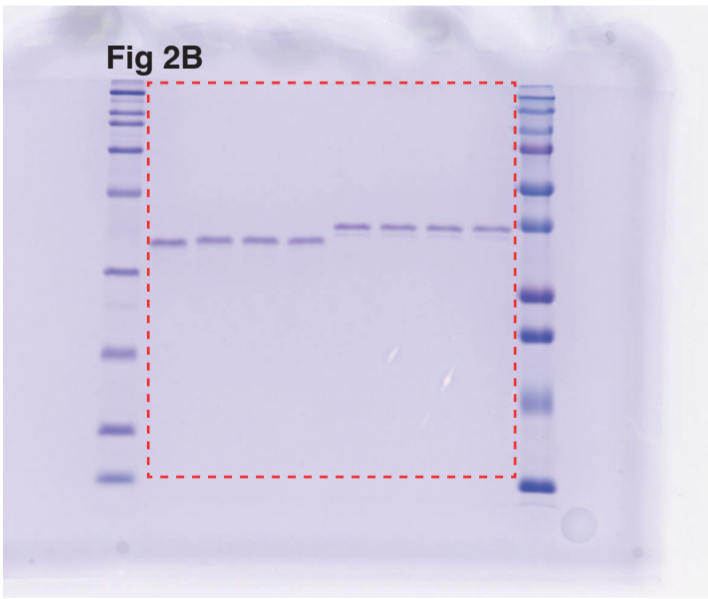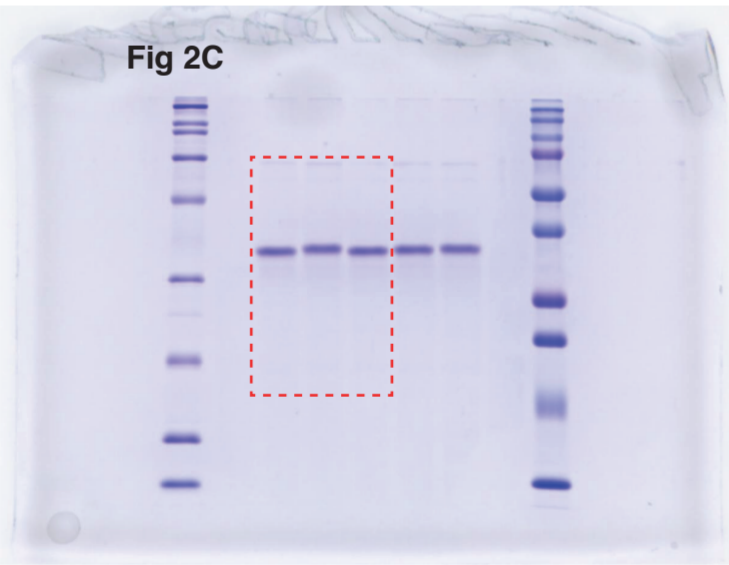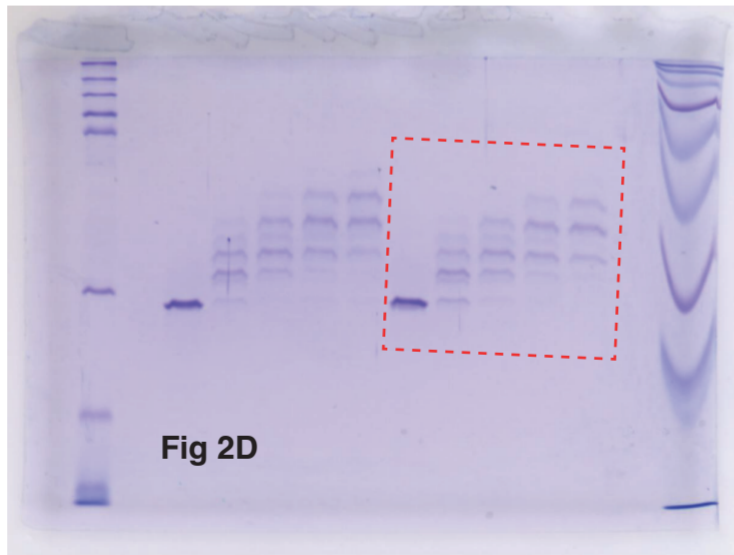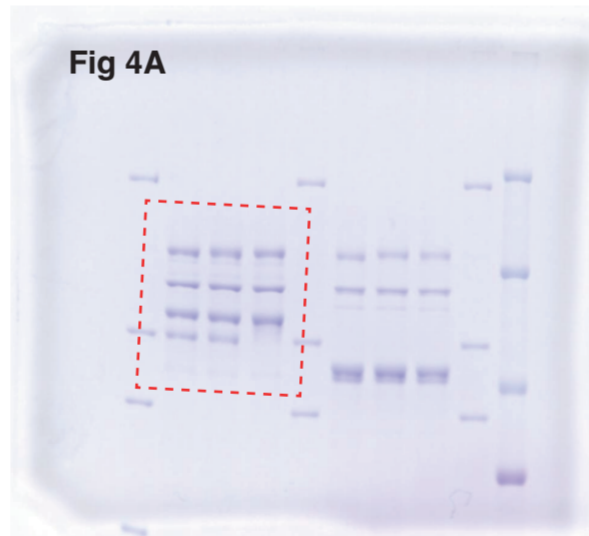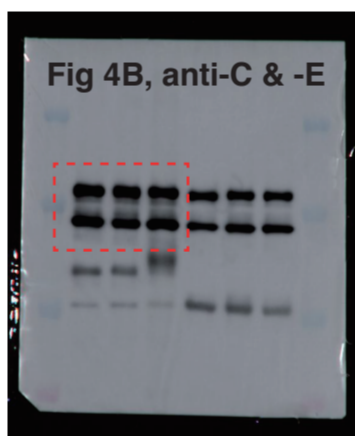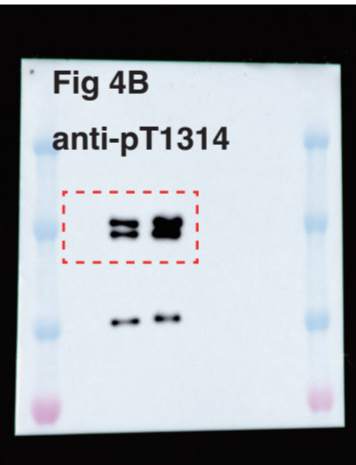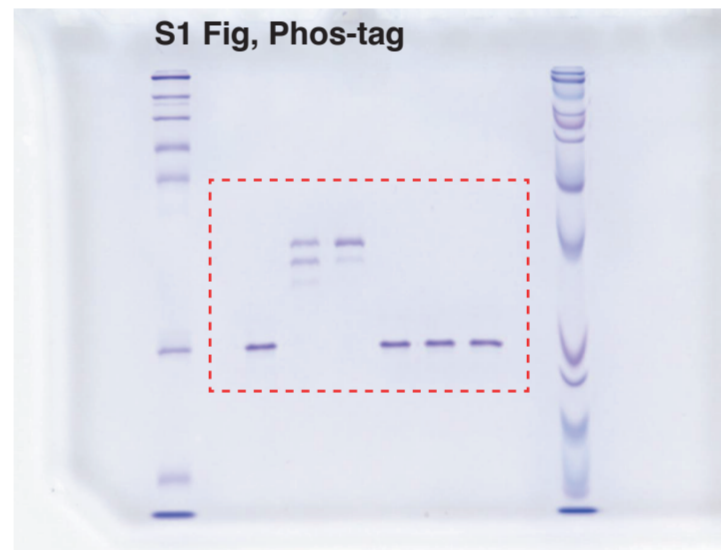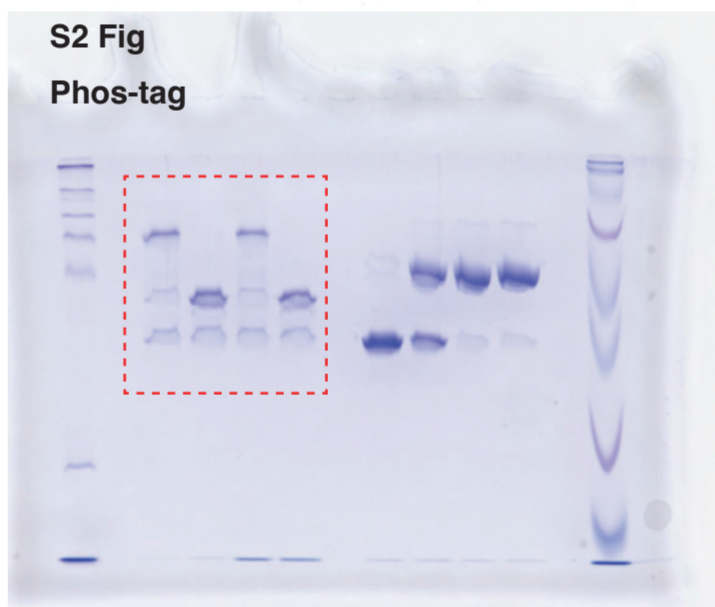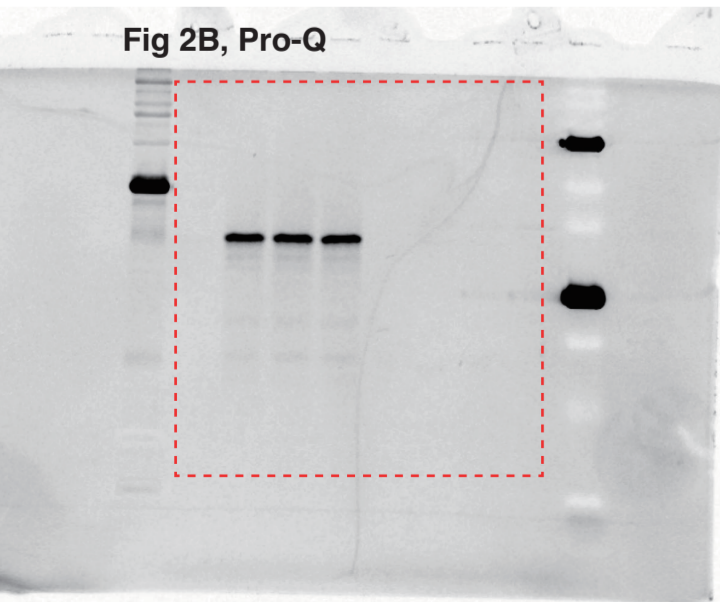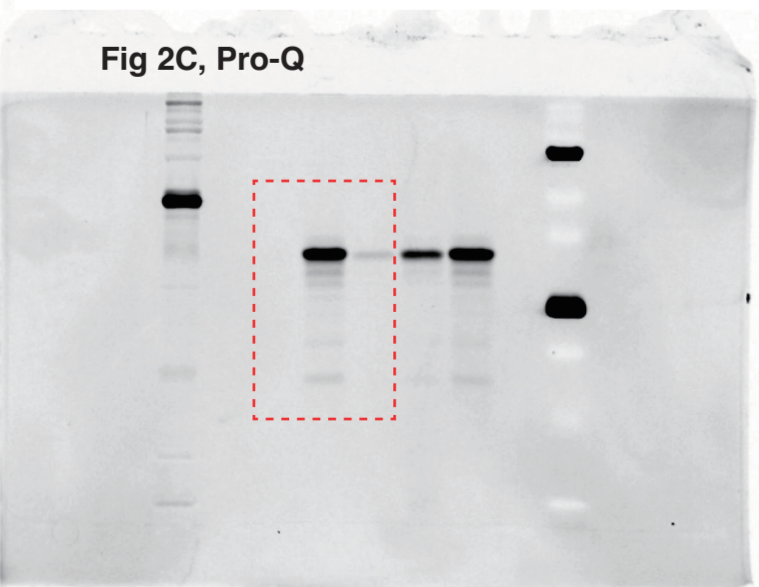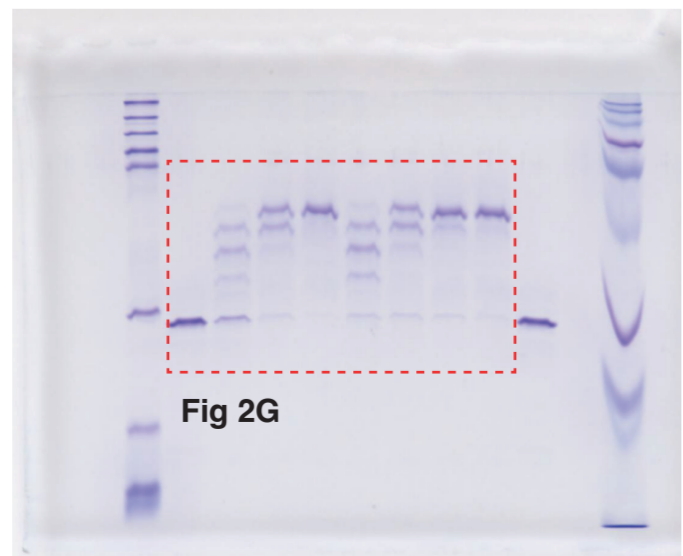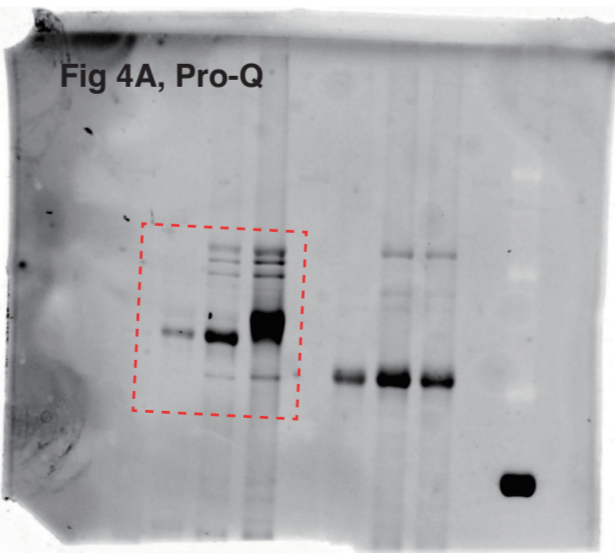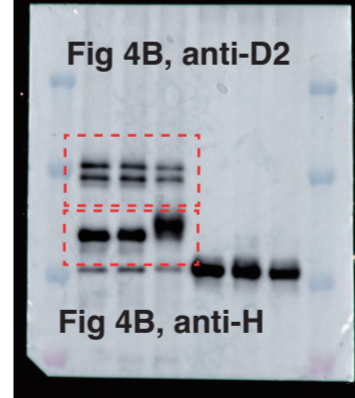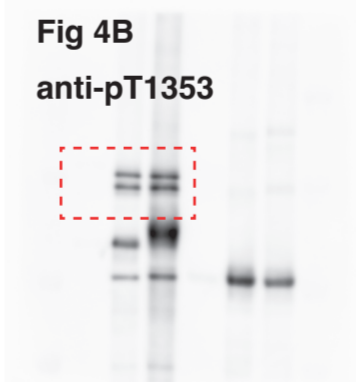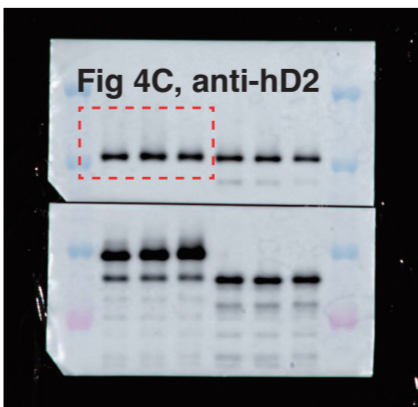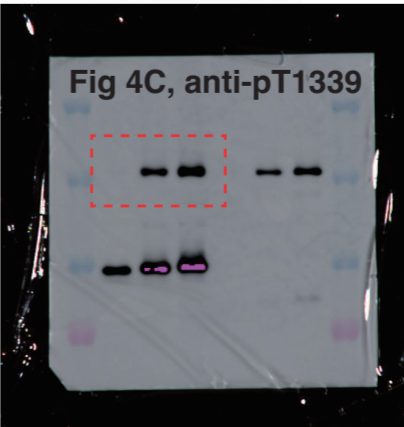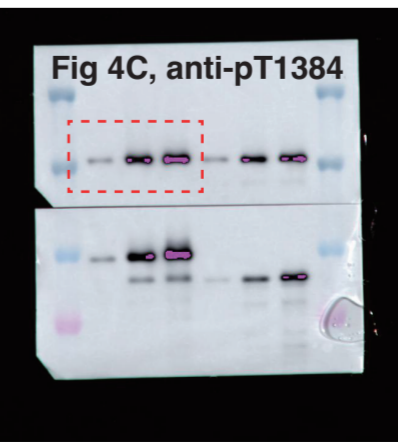

Supplement: S1 Raw images — (PDF) [file pone.0299003.s003.pdf]
